# Spatio-Temporal Mechanism Underlying the Effect of Urban Heat Island on Cardiovascular Diseases

**DOI:** 10.18502/ijph.v49i8.3889

**Published:** 2020-08

**Authors:** Huanchun HUANG, Xin DENG, Hailin YANG, Xinhui ZHOU, Qi JIA

**Affiliations:** 1. College of Landscape Architecture, Nanjing Forestry University, Nanjing, China; 2. College of Arts and Design, Zhengzhou University of Light Industry, Zhengzhou, China

**Keywords:** Urban heat island, Cardiovascular disease, Landscape pattern, Spatio-temporal characteristic

## Abstract

**Background:**

We explored the spatio-temporal characteristics of urban heat island (UHI) effect on cardiovascular diseases (CVDs).

**Methods:**

The land surface temperatures (LST) were retrieved from four Landsat remote-sensing images’ data, the temperature data from 95 meteorological stations, and analysis data on CVDs mortality. Based on these data, landscape pattern indexes were used to analyze the pattern-process-function and the mechanism.

**Results:**

During 1984–2017, the effects of UHI on CVDs increased, thereby increased the mortality by 28.8%. The affected areas gradually expand from the central area of the city and undergo three evolution stages; the highly affected areas are mainly distributed in central and southern regions, and patches increase in number. The areas and ratio of high-level patches also show an upward tendency, increasing dominance in the overall landscape. Patches of the overall landscape become more complicated in shape, whereas those of high-level ones become less complicated. Concentration degree of the overall landscape decreases gradually with the types of landscapes patches increasing, reaching a rather even space distribution.

**Conclusion:**

Increased temperatures exacerbated by UHI lead to increased CVD mortality. As cities expand, the effects of UHI on CVDs increase in terms of both intensity and areas, with the overall landscape in uneven distribution, high-level affected areas in point distribution, and low-level ones in large-area concentration.

## Introduction

Currently, 50% of the world population lives in cities, and this proportion is expected to increase to 68% by 2050. As people continue to migrate to cities, built-up areas keep on expanding, which make the temperature in urban areas significantly higher than that in the surrounding rural areas (1–2). This increased temperature adversely affects the health, particularly the cardiovascular system of urban residents.

Cardiovascular diseases (CVDs) are the leading cause of death worldwide. In 2016, altogether, 17.9 million people died from CVDs. Many factors cause CVDs and CVD-related deaths. At present, many studies have explored the relationship between air temperature and CVDs based on epidemiology (3).

The excess mortality in summer is attributed to an increased prevalence of CVDs (4–5). The exposure-response curve of temperature-CVDs mortality is usually depicted as U-, V-, or J-shaped, indicating that mortality increases gradually when the temperature exceeds the threshold (6). Threshold temperature, a critical standard for identifying the extent of urban heat island (UHI)-mediated damage on health (7–8), can help explore the spatio-temporal pattern characteristics of UHI’s effect on CVDs. Previous studies, however, mainly focused on quantifying the effect of environmental temperature on CVDs using epidemiology, which cannot explain the mechanism underlying the spatio-temporal interaction between them. Therefore, studies on the landscape pattern between UHI and CVD mortality are of critical significance.

Scholars have used the landscape pattern indexes to analyze the spatio-temporal evolution of heat island landscape (9–10), without any focus on the response mechanism of human health. In fact, landscape pattern is related to multiple diseases (11–12). Regional landscape fragmentation and temperature positively correlate to the incidence of Lyme disease (13). This indicates that landscape pattern is related to human health; however, the research focuses on the mechanism of landscape structure influencing disease, not on the landscape-pattern-process and its mechanism. From 1984 to 2018, the UHI effect has become more serious, and the incidence of heat wave has increased significantly, particularly in world-class megacities like Beijing, which has led to a sharp rise in CVD mortality. However, the spatio-temporal characteristics of UHI on CVD mortality remain unclear.

The study used data from Landsat remote-sensing and meteorological stations and maps and analyzed the spatio-temporal evolution of the landscape pattern of UHI on CVDs using data analysis software, such as ENVI, ArcGIS, and Fragstats.

## Materials and Methods

### Data Resources

Data on temperature were retrieved from Landsat satellite images and meteorological stations. The remote-sensing images were taken on August 16, 1984; June 17, 1991; August 2, 1999; and July 10, 2017. When the satellite was present in the Chinese territory, the atmospheric visibility was sound without cloud covering the study area, and the average wind speed 2 days before the image collection was below 2.3m/s without any precipitation. The meteorological data were collected via hourly observations from the 96 meteorological stations in Beijing.

CVDs (I00–I60) were classified according to the International Classification of Diseases, 10th revision (ICD-10). Data on CVD mortality in Beijing were obtained from the analysis of the Chinese Center for Disease Control and Prevention (14).

### Data Preprocessing

First, radiometric calibration and atmospheric correction were performed on remote-sensing images. The images were calibrated to 0.5 m resolution and any error was controlled within 1 pixel. Next, data were resampled to 30m resolution to be projected to the WGS_1984_ UTM_50N coordination system. Last, a database was established by the ArcGIS software to extract remote-sensing data in different periods for analysis.

### Research Methods

#### Temperature Retrieval

Land surface temperature (LST) and near-surface air temperature (NSAT), two critical parameters that reflect UHI’s interaction mechanism, are found to have a good correlation. Studies have proved that LST and land surface index by Thematic Mapper (TM) retrieved (15, 16) can serve as valid data to study urban thermal health (17).

Atmospheric calibration was applied in this study. First, radiometric calibration was performed according to the NASA manual (www.usgs.gov/landsat) to transform the digital number (DN) into relevant heat radiation intensity. Then, Normalized Difference Vegetation Index (NDVI) and vegetation coverage were calculated. Next, the calculation method for emissivity proposed by Qin (18) was used to obtain the land surface emissivity via NDVI and vegetation coverage. Finally, LST was calculated using the following (*T_L_*) formula:

$$T_{L}=273.15T/\left[1+\left(\lambda T/\rho \right)\text{ln}\varepsilon \right]$$

where (*λ*) is the central wavelength of TM6 band (11.5μm), *ρ* = *hc* /*σ* = 1.438·10^−2^ *K* (where Stefan-Boltzmann constant *σ* = 1.38·10^−23^ *J* / *K*, Planck constant *h* = 6.626 ·10^−34^ *Js*, and speed of light *c* = 2.998·10^8^*m* / *s*).

Data on LST retrieval were collected from the tests in 95 surface meteorological stations on July 10 and August 22, 2015, based on which the temperature regression formula was established. First, the coefficient between NDVI, Normalized Difference Water Index (NDWI), architectural composition, and daily average temperature was calculated on various scales. The results revealed that daily average temperature was closely related to NDVI at 160 m and LST at 300 m resolution. Next, software, such as SPSS and MATLAB, were used for curvilinear regression, indicating that linear polynomial enjoys sound regression results and that regression formulas boast sound robustness. The coefficient of determination (*R*^2^) was 0.95, the root-mean-square error was 0.13, and the regression formula is as follows:

$$T_{A}=4.31+0.1678T-0.1747y$$

where (*T_A_*) is the daily average temperature, (*T_L_*) is the LST, and (*y*) is the NDVI.

### Relationship between Increased UHI and Increasing CVD Mortality

**Fig. 1:**
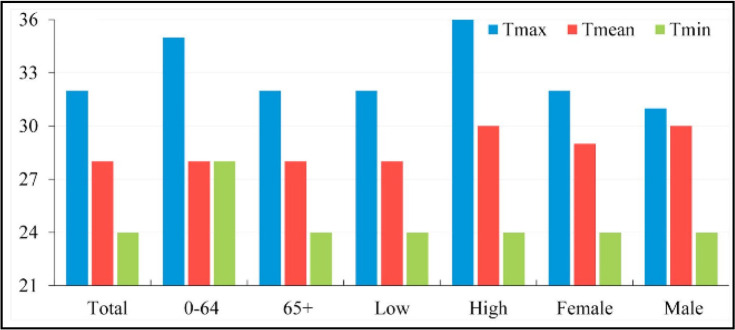
Heat thresholds of Tmax/Tmean/Tmin (12)

UHI drives the urban temperature, which leads to increased CVD mortality and morbidity, but the influence of high temperature on such mortality still faces a key threshold value (19–20). Based on daily temperature and CVD mortality data from 2007 to 2013 in Jinan City, China, Li estimated the thermal threshold of CVDs mortality using observations and prospective analysis. Studies have shown the maximum, average, and minimum threshold temperatures in Jinan are 32 °C, 28 °C, and 24 °C, and the mortality increases by 4.1%, 7.2%, and 6.6%, respectively, with every 1 °C increase in temperature, while in Beijing, the average threshold temperature was 25 °C–33 °C (14) (Fig. 1).

Because Beijing and Jinan are both temperate monsoon with small longitude difference and belong to the same longitude megacities, this study is based on the relationship between daily mean temperature and CVD mortality, to explore the spatio-temporal evolution characteristics of UHU on CVDs

### Grading of the Effect of UHI on CVDs

Modern medical studies have revealed that environmental temperature is closely related to the physiological activities of human body. The optimal environmental temperature is 20–28 °C. If the temperature exceeds 28 °C, our blood vessels dilate, sending more blood transports to the skin surface, thereby increasing the skin temperature and making us feel uncomfortable. Once the temperature exceeds 30 °C, the human body would activate some of our sweat glands, to emit heat out as perspiration, thereby dilatating the skin vessels and re-allocating the blood. At the same time, more blood comes to and out of the heart, aggravating the cardiac load (21–22). When the temperature goes beyond 32 °C, it reaches the maximum threshold temperature for CVD mortality in Jinan, China (14).

According to the threshold temperature and the physiological response of the human body to high temperature, this study is based on the threshold temperature at which UHI affects CVDs in Beijing with the daily average temperature of 28 °C, and the UHI intensity is 2 °C. With every 1 °C rise of threshold temperature, the mortality increases by 7.2%. Therefore, this study divided UHI’s influence on CVD mortality into five levels as shown in Table 1. Level 1 indicates that UHI has a lower effect on CVD. Levels 2, 3, 4, and 5 represent medium, high, and very high effects.

**Table 1:** Grading of UHI’s influence on CVD mortality

***Level***	***Temperature/°C***	***UHI intensity/°C***	***Mortality rise/%***	***Remark***
Level 1	28–29	2.7–3.7	0–7.2	Human starts to feel a little uncomfortable
Level 2	29–30	3.7–4.7	7.2–14.4	Human feels uncomfortable, with slight sweating
Level 3	30–31	4.7–5.7	14.4–21.6	Human feels very uncomfortable, with a lot of sweating
Level 4	31–32	5.7–6.7	21.6–28.8	Cardiovascular system starts to be affected, with increased cardiac output, aggravated cardiac load
Level 5	32+	6.7+	28.8–36	Temperature reaches the critical value of daily maximum temperature for CVD mortality

### Evaluation Methods of Landscape Pattern Indexes

Landscape pattern refers to the permutation of landscape patches of various sizes in the landscape space (23). Quantitative analysis could be performed on changes and differentiation characteristics of landscape patterns by introducing landscape pattern indexes. The indexes include three levels: patch, class, and landscape. This study, therefore, started from class level and landscape level, selected 10 landscape pattern indexes to perform quantitative description on the landscape pattern characteristics that yield an influence on CVDs. The indexes selected include class level—PD, cohesion (COHESION), and landscape shape index (LSI)—and landscape level: main patch area (AREA_MN), shape index (SHAPE_AM), aggregation index (AI), contagion (CONTAG), and Shannon diversity index (SHDI). Indexes of the class level demonstrate the quantity and structure of patch types at all levels, while indexes of the landscape level reflect the global feature of the study area.

### Survey of Research Areas

**Fig. 2:**
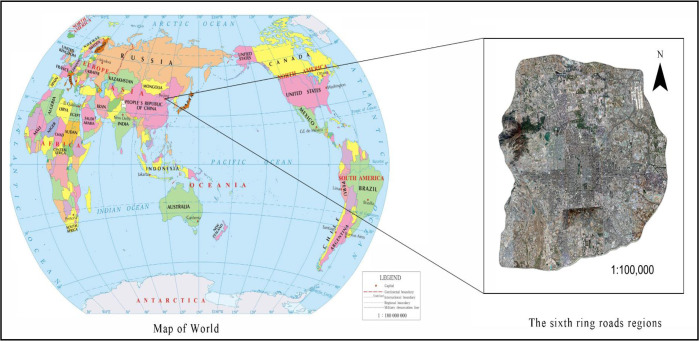
Study Area

Beijing (39.56°N, 116.20°E), one of the first-tier cities across the world, is the center of politics, economy, and culture of China, belonging to the semi-humid continental monsoon of the north temperate zone, which is hot and humid in summer (Fig. 2). At the end of 2018, permanent population in Beijing reached 21.542 million, the rate of urbanization was 86.5%, and the gross domestic product (GDP) exceeded 3 trillion yuan.

As the population and economy rocketed, environmental problems, such as UHI, arose, yielding a negative impact on both physiological and psychological health of urban residents. Against the backdrop of economic globalization, the urban sprawl of Beijing became more probable as the UHI became more severe (24); thus, high temperature engulfed Beijing for 22 days in 2017, catching more attention worldwide. It is therefore of significance to study UHI in Beijing.

## Results

### Spatial Characteristics of UHI’s Influence on CVDs

During 1984–2017, the influence of UHI on CVDs increased from level 2 to level 4, resulting in a 28.8% increase in mortality rate and a 1683.977 km^2^ increase in the affected area. Affected areas showed a trend of expanding from the central area outward in spatial distribution, showing an obvious spatio-temporal correlation with the process of urban sprawl. The highly affected areas were mainly concentrated in the central and southern parts, in that the air temperature in the northern mountainous area was relatively low, making it easier to build up high pressure. Meanwhile, air temperature in urban area was relatively high, resulting in low pressure and triggering cold air flowing from the northern mountainous area to the urban area.

**Fig. 3:**
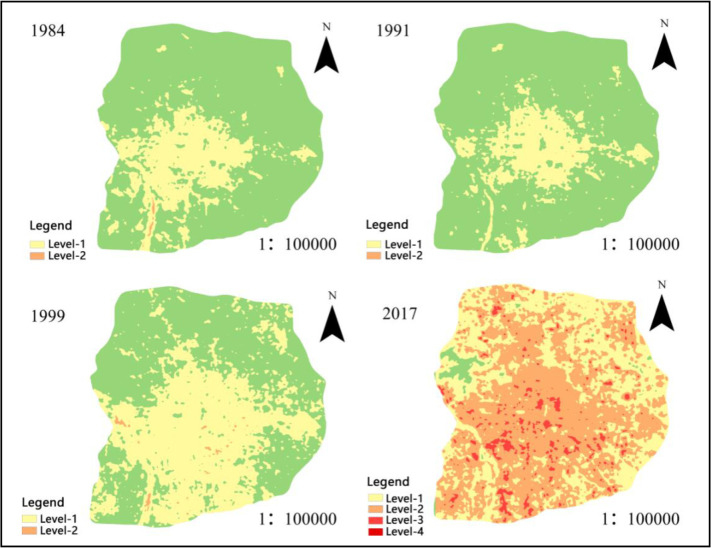
Distribution of affected areas during 1984–2017

As shown in Fig. 3, there only emerged level 1 and level 2 affected areas within the sixth ring road of Beijing during 1984–1999; no highly affected areas were spotted. In detail, level 1 affected areas were mainly distributed in the urban center, gradually expanding to the southern suburbs. Level 2 affected areas were smaller and scattered in the south. In 2017, level 1 patches drastically shrank, while level 2 had expanded drastically and transformed from scattered points to relatively concentrated plates and facets. Meanwhile, some of the low-level patches in business centers turned into high-level ones. Such drastic changes in the spatial distribution of all affected areas were triggered by the fact that in the past two decades, Beijing city (particularly the urban areas) developed rapidly as the business gathered, resulting in a series of environmental problems and affecting the cardiovascular health of residents.

### Landscape Pattern Analysis of UHI’s Influence on CVDs

#### Changes in Class Level

The changes in PD between 1984 and 2017 are shown in Table 2. In general, PD increased in a fluctuating manner, indicating that affected areas at all levels showed a tremendous fragmentation and separation trend. In detail, during 1984– 1991, level 1 and level 2 affected areas increased slowly yet stably due to the rather slow pace of urban construction. During 1999–2017, however, level 1 and level 2 affected areas decreased, while level 3 and level 4 affected areas increased. This was mainly because urban sprawl tremendously increased the temperature in urban centers, exacerbating the cardiovascular health of residents, leading to the gradual transformation of the level 1 affected area into high-level ones.

**Table 2:** Changes in PD during 1992–2018

***Variable***	***1984***	***1991***	***1999***	***2017***
Level 1	0.0692	0.078	0.1159	0.0957
Level 2	0.0022	0.0022	0.0159	0.071
Level 3	0	0	0	0.1275
Level 4	0	0	0	0.0004

The changes in LSI between 1984 and 2017 are shown in Table 3. LSI generally increased, indicating that affected areas at all levels became more complicated in shape. During 1999–2017, LSI of level 1 and level 2 affected areas increased rapidly, in that the fast urbanization triggered leapfrog expansion of urban shape and added many patches of independent built-up areas. Meanwhile, level 3 and level 4 affected areas were less complicated because high-level affected areas were mainly concentrated in business centers and residential areas where patch shapes tended to be simpler.

**Table 3:** Changes in LSI during 1992–2018

***Variable***	***1984***	***1991***	***1999***	***2017***
Level 1	12.1617	13.1989	14.5865	23.0332
Level 2	3.5948	2.3	6.8798	22.6149
Level 3	0	0	0	18.6593
Level 4	0	0	0	1.0714

The changes in COHESION between 1984 and 2017 are shown in Table 4. The COHESION of level 1affected areas were stable, indicating that patches shared sound interconnectivity with relatively concentrated distribution, which was not beneficial to the rapid thermal dissipation inside patches. The elevated air temperature increased CVD mortality. COHESION of level 2 affected areas fluctuated due to the urban renovation. In addition, COHESION of level 3 and level 4 affected areas was relatively low with weaker interconnectivity, in that patches were small in area and distributed in points.

**Table 4:** Changes in COHESION during 1992–2018

***Variable***	***1984***	***1991***	***1999***	***2017***
Level 1	99.8218	99.6738	99.8833	99.7612
Level 2	97.0525	90.0021	96.2389	99.9433
Level 3	0	0	0	96.9449
Level 4	0	0	0	84.978

### Changes in Landscape Level

**Fig. 4:**
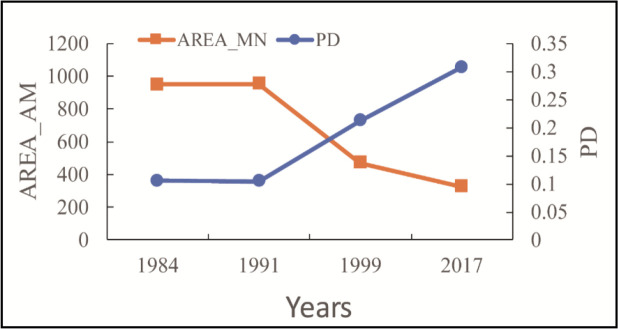
Changes in PD and AREA_MN during 1984–2017

The changes in PD and AREA_MN between 1984 and 2017 are shown in Fig. 4. PD generally increased, while AREA_MN index decreased, indicating that the landscape distribution was heading toward fragmentation.

**Fig. 5:**
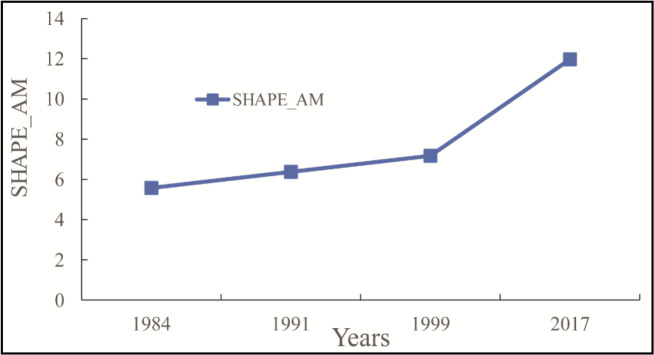
Changes in SHAPE_AM during 1984–2017

During 1984–1991, PD and AREA_MN nearly remained unchanged due to the rather slow pace of urban construction. The changes in SHAPE_AM between 1984 and 2017 are shown in Fig. 5. SHAPE_AM generally increased, indicating that the shape of all affected areas became irregular and that the effect of UHI on CVDs became more and more complicated in spatial distribution. During 1984–1999, SHAPE_AM increased steadily due to the rather slow pace of urban development and that urban form was quite stable. However, SHAPE_AM increased rapidly during 1999–2017 in that rapid urban development and three-dimensional expansion brought many new business centers and development projects.

**Fig. 6:**
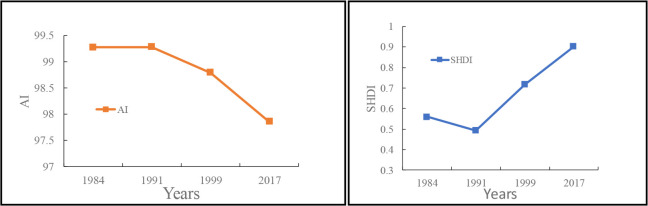
Changes in AI and SHDI during 1984–2017

The changes in AI and SHDI between 1984 and 2017 are shown in Fig. 6. AI decreased in general, indicating that the overall landscape was distributed in a scattered way, and the landscape was highly fragmented. Patch adjacency was much more decentralized, and the interconnectivity within the affected areas was weak. During 1984– 2017, SHDI, an index that describes patch diversity, generally increased, indicating that urban built-up areas expanded toward suburbs. In addition, the increased UHI yielded a greater impact on more types of landscape patches with a higher degree of fragmentation. During 1984–1991, SHDI decreased slightly, in that UHI’s influence on CVDs mortality was not very obvious as the urbanization slowed down, so that the expansion of level-2 areas dropped in scale, resulting in reduced diversity of patch types.

### Responding Mechanism of UHI on CVDs

High temperature increases the CVDs mortality by affecting the cardiopulmonary functions, including blood pressure, blood viscosity, serum cholesterol, and heart rate (25–26). First, elevated air temperatures increase the heart rate and myocardial oxygen consumption in the human body, meanwhile blood travels faster from organs to the skin surface, creating higher pressure on the heart and lungs (27), further increasing CVD incidence. In addition, the amount of perspiration from human body increases, leading to a massive loss of sodium, pH imbalance, and electrolyte disturbance in cells, further causing arrhythmia and malfunction of the circulatory system, which eventually induces CVDs (28). Moreover, blood viscosity and cholesterol level increase, but the effective circulating volume decreases, triggering insufficient blood supply via the coronary arteries and even myocardial infarction (29). Last, inappropriate use of air conditioning also indirectly increases CVDs mortality. In detail, when the indoor-outdoor temperature difference is >8 °C, it becomes difficult the for human body to adjust to the current temperature within a short time, and such drastic difference makes vessels continuously constrict or dilatate, resulting in a disturbance of blood circulation, and further triggering myocardial infarction or stroke.

UHI yields a more prominent impact on CVD mortality of elderly people aged over 65 by increases the temperature in summer. Under high temperature, the body functions of elderly people decline, and the amount and rate of perspiration of each gland reduce; thus, loss of heat in turn increases the accumulated heat inside the body, exacerbating the tension on the cardiovascular system. Under long-time thermal stimulus, constant perspiration causes the blood plasma volume to decrease dramatically; however, blood viscosity increases, thereby increasing CVD mortality (7). Moreover, some drugs usually taken by elderly people disturb the normal perspiration or other processes that adjust body temperature, thereby exacerbating CVD mortality.

Elevated temperature in summer caused by UHI yields a severe impact on CVDs, which is usually accompanied by increased air pollution. Atmospheric particulates triggered by heat waves increase in density and increase blood viscosity, pressure, and heart rate variability, thereby triggering diseases such as acute myocardial infarction (30). Moreover, the increase in ozone density caused by high temperature exacerbates the mortality of CVDs. Some studies have proved that with every 10μg·m^−3^ rise in ozone levels in the air, the mortality of CVDs among the Chinese population increases by 0.448% (31). This is because the increased ozone density triggers inflammation, oxidative stress, and myocardial cell damage and affects vessel structure and transcription mechanism, lipid metabolism, and cardiovascular autonomic regulation (32).

## Discussion

The study proves that UHIs have a significant effect on cardiovascular mortality by increasing ambient temperature in summer. Previous studies have proved that high temperatures significantly correlated with CVDs mortality (33). Maximum air temperature was significantly positively correlated to CVD mortality (r = 0.83, p = 0.01); the mortality increased 4.27% with every 1 °C rise in the maximum air temperature (95% confidence interval, 0.91–7.00) (34). This is consistent with our study. Moreover, related studies have explained the reasons. Heat waves activated the inflammation system, destroyed the structure of endothelial cells in coronary arteries, increased the permeability of the tunica intima, reduced the SOD activity of heart tissues, and finally increased the lipoproteins in the oxygenated blood. As a result, a large amount of cholesterol was accelerated and deposited on the tunica intima, causing atherosclerosis, thereby exacerbating the coronary heart disease (35). The above-mentioned studies focused on the influence of high temperature on CVDs based on threshold temperature or physiological mechanism, without analyzing the spatial distribution of high temperature and CVD mortality.

**Fig. 7:**
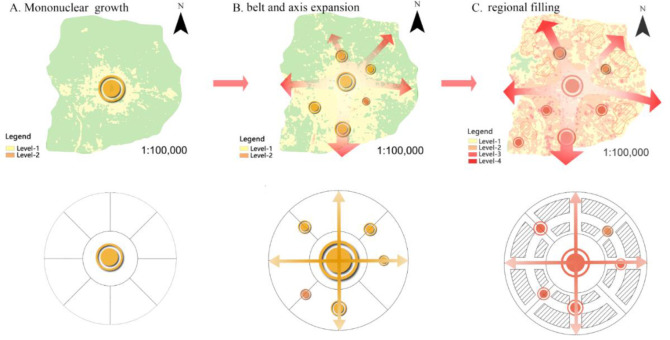
Stages of form evolution of UHI on CVDs

This study proves that the UHIs effects on CVDs have typical spatial and temporal characteristics, and the affected areas gradually expand to suburban and are mainly concentrated in the central and southern urban areas. Previous studies have reported that the high temperature clusters of heat wave in Beijing are mainly concentrated in the second ring road of Beijing, whereas heat wave risk obviously decreases from the second to sixth ring road (36–37). The spatial distribution of high-temperature heat waves has certain similarities with our study, which further supports the effects of UHI on CVDs. Meanwhile, our findings showed that the effects of UHI on CVDs evolve through three stages, namely, concentric ring growth, axis expansion, and regional filling (Fig. 7).

The study indicated that the effects of UHI on CVDs have typical pattern-process-function characteristics. Vector-borne Lyme disease incidence is significantly related to the degree of regional landscape fragmentation and temperature (13). Therefore, it is feasible to use the landscape pattern index method to study the effects of UHI on CVDs. This study provides a theoretical basis for the optimization of urban spatial structure and green space system. In the process of rapid urban development, according to the law of coordinated change between environment and residents’ health, the heat hazard sensitive space of UHI was predicted, and then the countermeasures for the controllable and adjusted planning factors are taken.

This study uses the landscape indexes to assess the effect of UHI on CVDs, demonstrating the trend of spatio-temporal evolution of influence, and providing theoretical reference for early warning of CVDs. However, this study had some limitations. One limitation is that we used the linear regression models; this study subtracted the effect of the intercept during air temperature retrieval to reflect the intensity of UHI better, resulting in overall underestimation on the effect on CVD mortality. Next, given the impact that weather and climate have on satellite images, this study used the two images to explore the spatio-temporal effect of UHI on CVDs during 1999–2017. Finally, the temperature-mortality relationship in this study cites the results of the Jinan study. The original study lacked data and did not consider air pollution, smoking, eating habits, etc., which may affect the temporal and spatial evolution of CVDs in UHI.

## Conclusion

As UHI keeps intensifying, the effect level and area on CVDs show an upward trend and three evolution stages (concentric ring growth, axis expansion, and regional filling). The affected areas spread from central urban area to suburbs, whereas more severe areas are mainly located in central and southern. The landscape pattern of the effect of UHI on CVDs reveals that, in terms of quantity, patches are gradually fragmented with the rise of patch quantity and density and high-level patches’ dominance. In terms of shape, the patches become irregular and space imbalanced; in terms of structure, the patch types of overall landscape are profuse yet weak in interconnectivity, whereas low-level areas are interconnected better in clusters.

## Ethical considerations

Ethical issues (including plagiarism, informed consent, misconduct, data fabrication and/or falsification, double publication and/or submission, and redundancy) have been completely observed by the authors.
